# Gas-Phase Internal Ribose Residue Loss from Mg-ATP and Mg-ADP Complexes: Experimental and Theoretical Evidence for Phosphate-Mg-Adenine Interaction

**DOI:** 10.1021/jasms.2c00071

**Published:** 2022-07-07

**Authors:** Magdalena Frańska, Olga Stȩżycka, Wojciech Jankowski, Marcin Hoffmann

**Affiliations:** 1 Institute of Chemistry and Technical Electrochemistry, Poznań University of Technology, Berdychowo 4, 60-965 Poznań, Poland; 2 Faculty of Chemistry, Adam Mickiewicz University, Uniwersytetu Poznańskiego 8, 61-614 Poznań, Poland

**Keywords:** adenosine-5′-triphosphate, adenosine-5′-diphosphate, collision-induced dissociation, magnesium complexes, fragmentation pathway

## Abstract

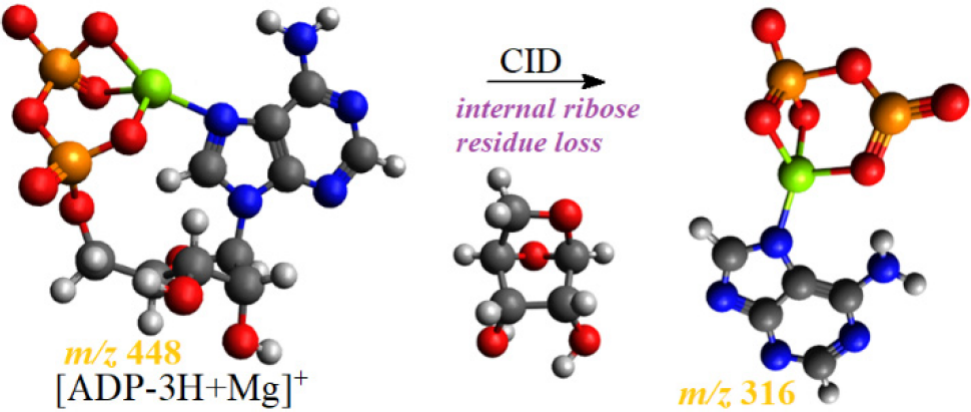

Gas-phase decompositions of magnesium complexes with adenosine-5′-triphosphate (ATP) and adenosine-5′-diphosphate (ADP) were studied by using electrospray ionization-collision-induced dissociation-tandem mass spectrometry, in the negative ion mode. The loss of internal ribose residue was observed and was found to occur directly from the [ADP-3H+Mg]^−^ ion. The occurrence of this process indicates the presence of a strong phosphate-Mg-adenine interaction. The performed quantum mechanics calculations confirmed the occurrence of this interaction in the [ADP-3H+Mg]^−^ ion, namely the presence of Mg–N7 bond and hydrogen bond between the phosphate oxygen atom and amino group. Although the finding concerns the gas phase, it indicates that phosphate-Mg-adenine interaction may be also of importance for biological processes. The loss of an internal ribose residue was also observed for calcium and zinc complexes with ATP/ADP as well as for magnesium complexes with guanosine-5′-triphosphate (GTP) or guanosine-5′-diphosphate (GDP). Therefore, it is reasonable to conclude that the presence of the phosphate-metal-nucleobase interaction is a feature of gas phase [NDP-3H+metal]^−^ ion (NDP, nucleoside-5′-diphosphate) and may also be important for biological processes.

## Introduction

All biological processes involving adenosine-5′-triphosphate (ATP) occur with the participation of magnesium ions, which has prompted extensive studies of the Mg-ATP complex by a number of techniques, for example, calorimetric titration,^1^ molecular dynamic,^2,3^ ab initio molecular orbital calculations,^4^ and mainly by NMR.^5−9^ Mass spectrometric techniques have been widely applied for the analysis of nucleotides, including ATP;^10−19^ however, to the best of our knowledge, there are no published data concerning the mass spectrometric investigation of the Mg-ATP complex. Therefore, we decided to determine the fragmentation pathways of Mg-ATP complex by using electrospray ionization-collision-induced dissociation-tandem mass spectrometry (ESI-CID-MS/MS). As described further, the unexpected loss of internal ribose residue was observed in negative ion mode (the loss of mass 132)^20^ for Mg-ATP and Mg-ADP complexes. Internal residue loss (the loss of the internal part of the fragmented ion) involving skeletal rearrangement is a quite common phenomenon for glyconjugates,^21−26^ but is not limited to them.^27−32^ From the analytical point of view, the occurrence of internal residue losses may be undesirable since it may hamper the structural elucidation, and this is why these processes are worth studying. Furthermore, the internal residue losses are some of the most interesting processes in the gas-phase ion chemistry. Internal ribose residue loss found in this work indicates that in the gas phase, we deal with strong phosphate-Mg-adenine interaction, which was supported by quantum mechanics calculations.

## Experimental Section

The sample solutions contained the nucleotide and respective chloride or nitrate in the concentration of about 5 × 10^–5^ M in methanol/water (1/1). In order to generate the abundant ions of interest, for example, [ATP-3H+Mg]^−^, the metal salt excess or nucleotide excess was used, for example, ATP concentration of 2 × 10^–5^ M and MgCl_2_ concentration of 6 × 10^–5^ M or ADP concentration of 6 × 10^–5^ M and Mg(NO_3_)_2_ concentration of 2 × 10^–5^ M. When the nucleotide excess was used, the abundant [nucleotide-H]^−^ ion was observed, and when the metal salt excess was used, the abundant [MgCl_3_]^−^ or [Mg(NO_3_)_3_]^−^ was observed (exemplary full scan mass spectra are shown in the Supporting Information, Figure S1).

The mass spectra were taken on a Waters/Micromass (Manchester, UK) ESI Q-tof Premier mass spectrometer (software MassLynx V4.1, Manchester, UK). The sample solutions were infused into the ESI source by a syringe pump at a flow rate of 5 μL/min. The electrospray voltage was 2.7 kV, and the cone voltage was 30 V, unless indicated otherwise. The source temperature was 80 °C, and the desolvation temperature was 250 °C. Nitrogen was used as the cone gas and desolvating gas at the flow rates of 0.8 and 13 L/min, respectively. Collision energy (CE) is the most important parameter for CID-MS/MS experiments, and it is indicated in each product ion spectrum shown. Argon was used as a collision gas at the flow rate of 0.2 mL/min in the T-wave collision cell.

### Computational

Energy calculations were performed, within the density functional theory framework at the M052X/Aug-ccPVTZ level of theory,^33−35^ which are recommended for noncovalent interactions by Goerigk et al.^36^ Counterpoise correction was calculated to assess basis set superposition error (BSSE) and to calculate interaction energy between Mg^2+^ and ADP structure.^37,38^ All quantum mechanics calculations were performed with Gaussian 09 available within Pl-Grid infrastructure.^39^

## Results and Discussion

**Figure 1:**
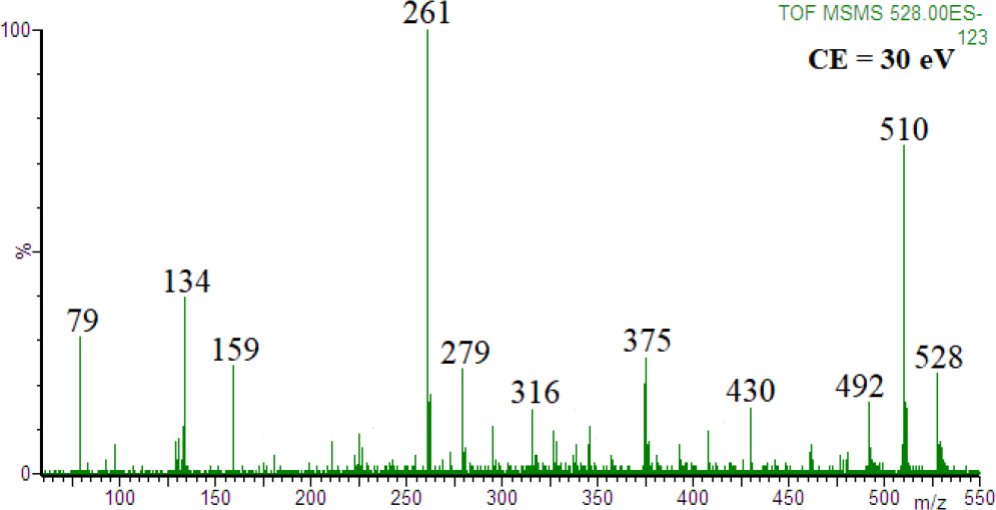
Product ion spectrum of [ATP-3H+Mg]^−^ ion, *m*/*z* 528; HPO_3_ and ribose residue loss, *m***/***z* 316.

Figure 1 shows the product ion spectrum of [ATP-3H+Mg]^−^ ion at *m*/*z* 528 (in the negative ion mode, this is the simplest Mg-ATP complex) obtained at the collision energy of 30 eV (the spectra obtained at lower collision energies are shown in the Supporting Information, Figure S2).

**Figure 2:**
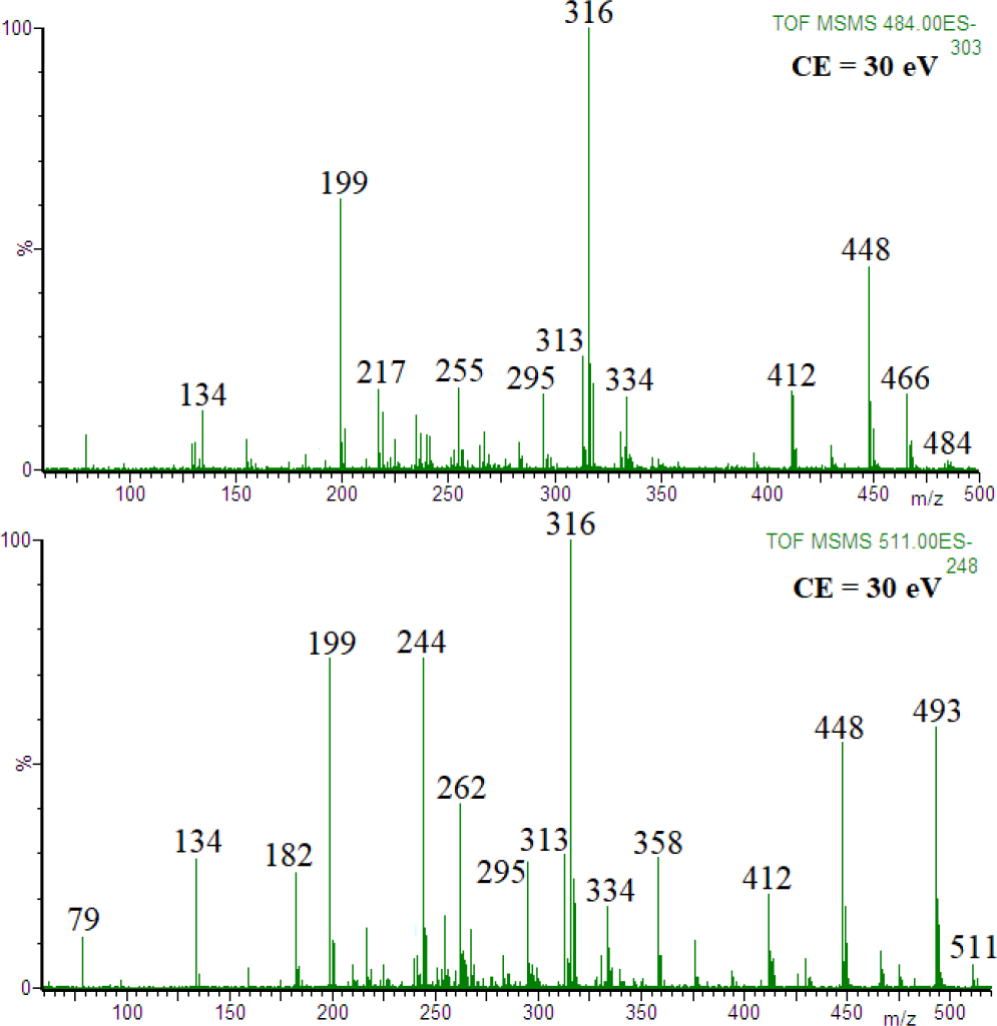
Product ion spectra of ions [ADP-2H+MgCl]^−^, *m*/*z* 484) and [ADP-2H+MgNO_3_]^−^ ion, *m*/*z* 511; HCl/HNO_3_ and ribose residue loss, *m***/***z* 316.

The measured accurate masses of product ions (Table 1, the product ions with relative abundances higher than 10% were taken into account) allowed easy determination of fragmentation pathways of the [ATP-3H+Mg]^−^ ion. For example, the most abundant product ions at *m*/*z* 261 and *m*/*z* 510 were formed by the loss of the adenosine molecule and water molecule, respectively, and the product ion at *m*/*z* 159 corresponds to the hydrogen dimetaphosphate anion,^40^ etc. The only product ion that was difficult to rationalize was that at *m*/*z* 316. Its elemental composition (Table 1) indicated that this ion was formed by the loss of HPO_3_ molecule (loss of mass 80) and ribose residue (loss of mass 132) from the [ATP-3H+Mg]^−^ ion. The loss of HPO_3_ molecule may be regarded as a trivial process, and formally it leads to the formation of ADP from ATP. The loss of ribose residue is not a trivial process, since it is an example of internal residue loss. It is reasonable that upon the loss of the HPO_3_ molecule, the product ion at *m*/*z* 448 was formed, which was not detected since it immediately underwent ribose residue loss producing the ion [(O_2_POPO_3_)Mg(adenine-H]^−^ at *m*/*z* 316. Thus, formally, the loss of internal ribose residue occurs for the Mg-ADP complex. Therefore, we obtained the respective product ion spectra for Mg-ADP complexes. However, the [ADP-3H+Mg]^−^ ion was not formed in the solutions containing ADP and magnesium salt. The ions containing the counterions were formed, namely [ADP-2H+MgCl]^−^ or [ADP-2H+MgNO_3_]^−^ (*m*/*z* 484 and *m*/*z* 511, respectively). Figure 2 shows the product ion spectra of these ions obtained at the collision energy of 30 eV (the spectra obtained at lower collision energies are shown in the Supporting Information, Figures S3 and S4).

**Table 1:** Results of Accurate Mass Measurements Obtained for Ions Detected from the Product Ion Spectrum of [ATP-3H+Mg]^−^ Ion

Composition	Exact mass	Measured mass	Error (ppm)
C_10_H_13_N_5_O_13_P_3_Mg	527.9573	527.9594	4.0
C_10_H_11_N_5_O_12_P_3_Mg	509.9468	509.9491	4.5
C_10_H_9_N_5_O_11_P_3_Mg	491.9362	491.9344	–3.7
C_10_H_10_N_5_O_9_P_2_Mg	429.9804	429.9819	3.5
C_5_H_6_O_12_P_3_Mg	374.8923	374.8907	–4.3
C_5_H_4_N_5_O_6_P_2_Mg	315.9487	315.9472	–4.7
H_2_P_3_O_10_Mg	278.8711	278.8727	5.7
P_3_O_9_Mg	260.8606	260.8612	2.3
HP_2_O_6_	158.9248	158.9236	−7.5
C_5_H_4_N_5_	134.0467	134.0461	4.5
PO_3_	78.9585	78.9582	–3.8

For both ions, the most abundant product ion is [(O_2_POPO_3_)Mg(adenine-H)]^−^ at *m*/*z* 316. It is reasonable that the product ion at *m*/*z* 316 was formed from that at *m*/*z* 448, as a result of ribose residue loss (loss of mass 132), whereas the product ion at *m*/*z* 448 was formed from the ion [ADP-2H+MgCl]^−^ by the loss of HCl (or by the loss of HNO_3_ from [ADP-2H+MgNO_3_]^−^). The product ion spectra of [ADP-2H+MgCl]^−^ obtained at lower collision energies confirm the above assignment, as shown in the Supporting Information (Figures S3 and S4).

The [ADP-3H+Mg]^−^ ion at *m*/*z* 448 was not formed in the solutions containing ADP and magnesium salt; however, it was formed in the gas phase by using high cone voltage (fragmentation in-source). Thus, it was possible to obtain its product ion spectra. As shown in the Supporting Information, the abundant product at *m*/*z* 316 was formed from the ion at *m*/*z* 448 (Figure S5 and S6).

**Scheme 1:**
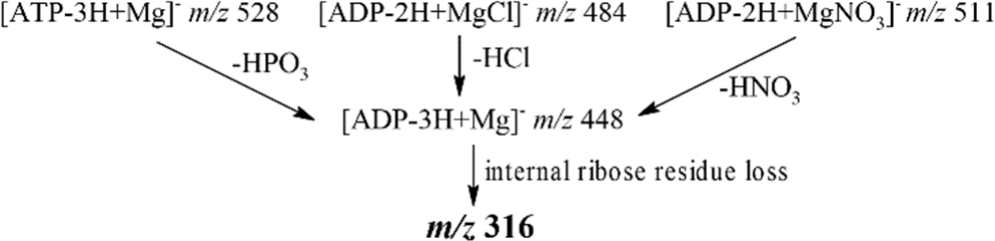
Fragmentation Pathways Leading to the Formation of the Product Ion at *m/z* 316

As clearly resulting from the above discussion, the internal ribose residue loss is preceded by the loss of the respective small molecules, as summarized in Scheme 1, and occurs directly from the [ADP-3H+Mg]^−^ ion.

No internal ribose residue loss was observed for the Mg-AMP complex, as indicated by the respective mass spectra shown in the Supporting Information (Figure S7). According to the published mass spectra, no internal ribose residue loss has been observed for the ions [ATP-2H+Na]^−^ and [ATP-H+Mg]^+^ (positive ion) and [nucleotide-H]^−^.^11,40−42^ Thus, the conclusion can be drawn that the internal ribose residue loss is a feature characteristic of negatively charged ATP-Mg and ADP-Mg complexes.

We also checked if the internal ribose residue loss occurs for ATP/ADP complexes with other metals, namely with calcium and zinc, which seem to be similar to magnesium (taking into account the chemical properties). Interestingly, the ADP noncounter ion-containing complexes were detected ([ADP-3H+Ca/Zn]^−^ ions) in the full ESI scan mass spectra (since analogical ions for Mg-ADP complex were observed only in the product ion spectra or at higher cone voltage, Figure 2 and Figure S5). As shown in the Supporting Information (Figures S8 and S9), the internal ribose residue loss took place, producing ion [(O_2_POPO_3_)Ca(adenine-H)]^−^ at *m*/*z* 332 and ion [(O_2_POPO_3_)Zn(adenine-H)]^−^ at *m*/*z* 356.

Guanosine-5′-triphosphate (GTP) seems to be the second most important in nature nucleoside triphosphate, and analogically as for ATP, the biological processes involving GTP occur with the participation of magnesium ions.^43−45^ Thus, GTP/GDP-Mg complexes were also included in the study. As shown in the Supporting Information (Figures S10 and S11), the internal ribose residue loss was observed, producing ion [(O_2_POPO_3_)Mg(guanine-H)]^−^ at *m*/*z* 332.

We also performed MS/MS experiments for the product ions formed as a result of internal ribose residue loss (it was possible since the product ions were generated by using high cone voltage, Figure S5). The obtained product ion spectra are shown and briefly discussed in the Supporting Information (Figure S12).

**Figure 3:**
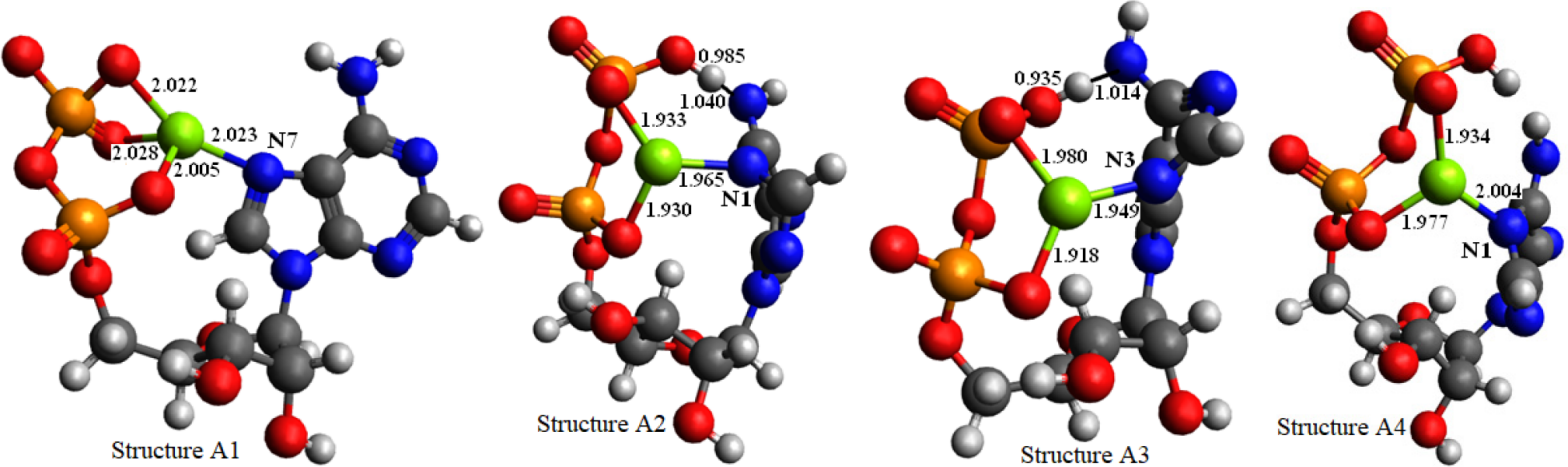
Optimized structures of [ADP-3H+Mg]^−^ ions with depicted bond length between coordination atoms and the central Mg^2+^ atom and the length of hydrogen bonds.

The key question is, in the [ADP-3H+Mg]^−^ ion, if we deal with Mg-adenine interaction (besides the obvious interaction between Mg^2+^ and deprotonated diphosphate moiety). The abundance of the product ion at *m*/*z* 316 (Figure 2) indicates that the existence of the Mg-adenine interaction is reasonable, most probably through the N7 atom.^46−50^ We considered four structures of [ADP-3H+Mg]^−^ ion, namely structure A1, containing the N7–Mg bond, structure A2, containing the N1–Mg bond, structure A3, containing the N3–Mg bond, and structure A4, containing the N1–Mg (complex of deprotonated imine tautomer). The obtained structures are shown in Figure 3, and the relevant atomic coordinates are shown in Tables S1 and S2 (Supporting Information). The calculated energies and interaction energies are shown in Table 2. By the interaction energy of the [ADP-3H+Mg]^−^ ion, we mean the difference between the sum of the energies of ADP^3–^ and Mg^2+^ and the energy of [ADP-3H+Mg]^−^ with included BSSE.

**Table 2:** Calculated Energies^a^ for the [ADP-3H+Mg]^−^ Ion

Structure	Counterpoise corrected energy (hartree)	BSSE energy (hartree)	Sum of monomers (hartree)	Interaction energy (uncorrected) (kcal/mol)	Interaction energy (corrected) (kcal/mol)
A1	–2297.349886	0.003950	–2296.214113	–715.2	–712.7
A2	–2296.769301	0.002850	–2295.776492	–624.8	–623.0
A3	–2296.711494	0.003597	–2295.687445	–644.9	–642.6
A4	–2296.935924	0.002519	–2295.912423	–643.8	–642.3

a Counterpoise energy, BSSE, sum of monomers energy, counterpoise uncorrected and corrected interaction energies.

The most stable was found to be structure A1 which contains the Mg–N7 bond. The obtained length of the Mg–N7 bond indicates that the bond is quite strong. Although the conformation change often occurs during the activation processes, it is reasonable to suppose that there is a strong phosphate-Mg-adenine interaction in the [ADP-3H+Mg]^−^ ion. As a consequence, in the CID conditions, the breaking of N-glycosidic bond and phosphoester bond (most probably heterolytic cleavage) leads to the internal ribose residue loss.

**Figure 4:**
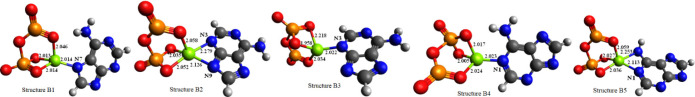
Optimized structures of [(O_2_POPO_3_)Mg(adenine-H)]^−^ ions with depicted bond lengths between coordination atoms and the central Mg^2+^ atom.

We also performed calculations for the structures of ion [(O_2_POPO_3_)Mg(adenine-H)]^−^. As it is possible that upon the fragmentation process the rearrangement may occur, coordination of Mg^2+^ by each of the adenine nitrogen atom was considered (five structures). The obtained structures are shown in Figure 4, the calculated energies and interaction energies (the difference between the sum of energies of O_2_POPO_3_^2–^, [adenine-H]^−^ and Mg^2+^ and the energy of [(O_2_POPO_3_)Mg(adenine-H)]^−^ with included BSSE) are shown in Table 3, and the relevant atomic coordinates are shown in Tables S3 and S4 (Supporting Information). The structure B5 was found to be the most stable, in which the Mg atom is coordinated by N1 and amino group, although the structure B1 which contains the Mg–N7 bond had very similar stability.

**Table 3:** Calculated Energies^a^ for the [(O_2_POPO_3_)Mg(adenine-H)]^−^ Ion

Structure	Counterpoise corrected energy (hartree)	BSSE energy (hartree)	Sum of monomers (hartree)	Interaction energy (uncorrected) (kcal/mol)	Interaction energy (corrected) (kcal/mol)
B1	–1801.312695	0.003538	–1800.223175	–685.9	–683.7
B2	–1801.032242	0.003751	–1800.009888	–643.9	–641.5
B3	–1794.690942	0.003774	–1793.629167	–668.6	–666.3
B4	–1801.301650	0.003725	–1800.222289	–679.7	–677.3
B5	–1801.303863	0.003719	–1800.209420	–689.1	–686.8

a Counterpoise energy, BSSE, sum of monomers energy, counterpoise uncorrected and corrected interaction energies.

**Scheme 2:**
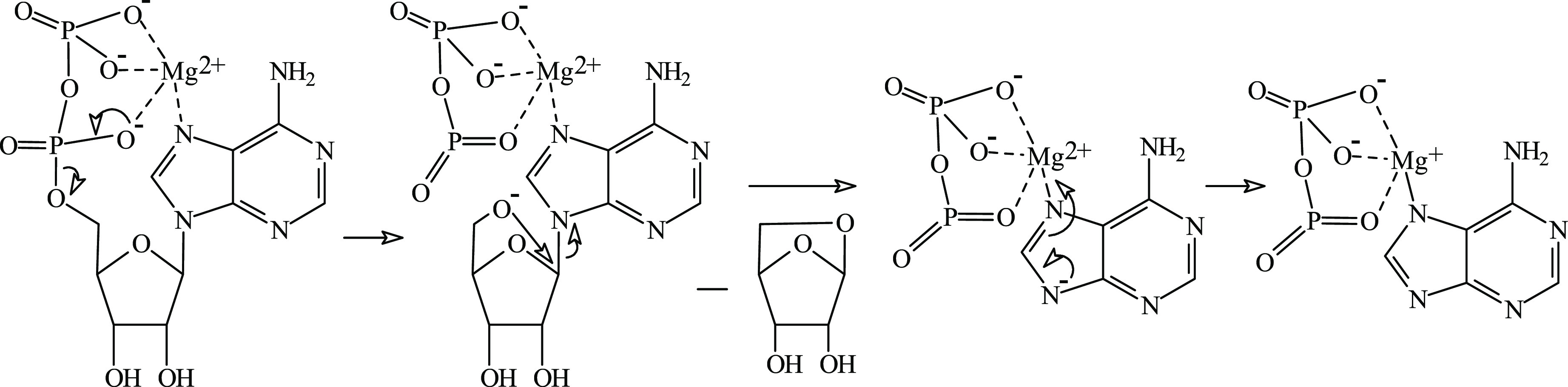
Plausible Mechanism of Internal Ribose Residue Loss

On the basis of the calculation results, we proposed a plausible mechanism of the observed internal ribose residue loss (Scheme 2). Namely, it was proposed that the loss of 1,5-anhydro-β-d-ribofuranose (atomic coordinates are shown in Table S5) from structure A1 of [ADP-3H+Mg]^−^ ion yields the [(O_2_POPO_3_)Mg(adenine-H)]^−^ ion of structure B1 (both ion structures contain Mg–N7 bond). Since the calculated counterpoise corrected energy of 1,5-anhydro-β-d-ribofuranose was −496,236719 hartree, the process shown in Scheme 2 was found to be energetically favored (−0.199528 hartree). Of course, further conversion of structures B1–B5 cannot be excluded. On the other hand, an analogical mechanism to that shown in Scheme 2 can be proposed to form the structure B5 from structures A2/A4 (B5 and A2/A4 contain Mg–N1 bonds). However, structures A2/A4 are less stable than A1 (Table 2); therefore, the occurrence of such process seems to be unlikely.

## Conclusions

By using ESI-CID-MS/MS and quantum mechanics calculation, it was found that in the [ADP-3H+Mg]^−^ complex, we deal with the phosphate-Mg-adenine interaction in the gas phase. Analogical interactions occur in the [GDP-3H+Mg]^−^ and [ADP-3H+Ca/Zn]^−^ complexes. Although the finding is interesting with respect to the gas-phase ion chemistry, the key question is if this interaction may also occur under physiological conditions. Gas-phase conditions are different from physiological conditions. In the latter, the solvent and counterion may affect the formation of the Mg–N7 bond and/or hydrogen bond. On the other hand, assuming that the gas-phase processes allow some insight into the biological processes, our finding indicates that the phosphate-Mg-adenine interactions in the Mg-ADP complex may be of importance for biological processes. Under physiological conditions, the interaction may be elusive, due to the presence of the solvent and counterions, which is why the interaction has not been detected yet. However, it is well-known that even very weak interactions are sometimes of crucial importance for biological processes.

## Supporting Information Available

Exemplary full scan mass spectra, a number of additional product ion spectra of Mg-ATP and Mg-ADP complexes (obtained by using different collision energies), product ion spectra of other metal-nucleoside-5′-tri- and diphosphates, atomic coordinates (PDF)
