# Supplementary material for: Gas-Phase Internal Ribose Residue Loss from Mg-ATP and Mg-ADP Complexes: Experimental and Theoretical Evidence for Phosphate-Mg-Adenine Interaction
Source: J Am Soc Mass Spectrom. 2022 Jul 7;33(8):1474–9. doi: 10.1021/jasms.2c00071 (PMC9354248; doi:10.1021/jasms.2c00071)
Supplement: Supplementary file 1 — js2c00071_si_001.pdf [file js2c00071_si_001.pdf]

Supporting Information

Gas-phase internal ribose residue loss from Mg-ATP and Mg-ADP complexes – experimental and theoretical evidence for phosphate-Mg-adenine interaction

Magdalena Frańska*[1], Olga Stężycka[1], Wojciech Jankowski[2] and Marcin Hoffmann[2]

[1] - Institute of Chemistry and Technical Electrochemistry, Poznań University of Technology, Berdychowo 4, 60-965 Poznań, Poland. Email: magdalena.franska@put.poznan.pl

[2] - Adam Mickiewicz University, Faculty of Chemistry, Uniwersytetu Poznańskiego 8, 61-614 Poznań, Poland.

Table of contents:

Figure S1. Exemplary full scan ESI mass spectra obtained for methanol/water solutions containing nucleotide and $MgCl_2$ or $Mg(NO_3)_2$.

Figure S2. Product ion spectra of $[ATP\text{-}3H+Mg]^-$ ion – *m/z* 528.

Figure S3. Product ion spectra of $[ADP\text{-}2H+MgCl]^-$ ion – *m/z* 484.

Figure S4. Product ion spectra of $[ADP\text{-}2H+MgNO_3]^-$ ion – *m/z* 511.

Figure S5. Full scan ESI mass spectra obtained for solution containing ADP and $MgCl_2$ at different cone voltages (CV).

Figure S6. Product ion spectra of $[ADP\text{-}3H+Mg]^-$ ion – *m/z* 448 (ion generated at high cone voltage).

Figure S7. Product ion spectra of $[AMP\text{-}2H+MgCl]^-$ ion – *m/z* 404 and $[AMP\text{-}2H+MgNO_3]^-$ ion – *m/z* 431.

Figure S8. Product ion spectra of $[ATP\text{-}3H+Ca]^-$ ion – *m/z* 544 and $[ATP\text{-}3H+Zn]^-$ ion – *m/z* 568.

Figure S9. Product ion spectra of $[ADP\text{-}3H+Ca]^-$ ion – *m/z* 464 and $[ADP\text{-}3H+Zn]^-$ ion – *m/z* 488.

Figure S10. Product ion spectra of $[GTP\text{-}3H+Mg]^-$ ion – *m/z* 544.

Figure S11. Product ion spectra of $[GDP\text{-}3H+Mg]^-$ ion – *m/z* 464.

Figure S12. Product ion spectra of product ions formed as a results of internal ribose residue loss from studied complexes.

Table S1. Atomic coordinates of structures A1 and A2 of $[ADP\text{-}3H+Mg]^-$ ion.

Table S2. Atomic coordinates of structures A3 and A4 of $[ADP\text{-}3H+Mg]^-$ ion.

Table S3. Atomic coordinates of structures B1, B2 and B3 of $[(O_2POPO_3)Mg(adenine\text{-}H)]^-$ ion.

Table S4. Atomic coordinates of structure B4 and B5 of $[(O_2POPO_3)Mg(adenine\text{-}H)]^-$ ion.

Table S5. Atomic coordinates of 1,5-anhydro-β-D-ribofuranose.

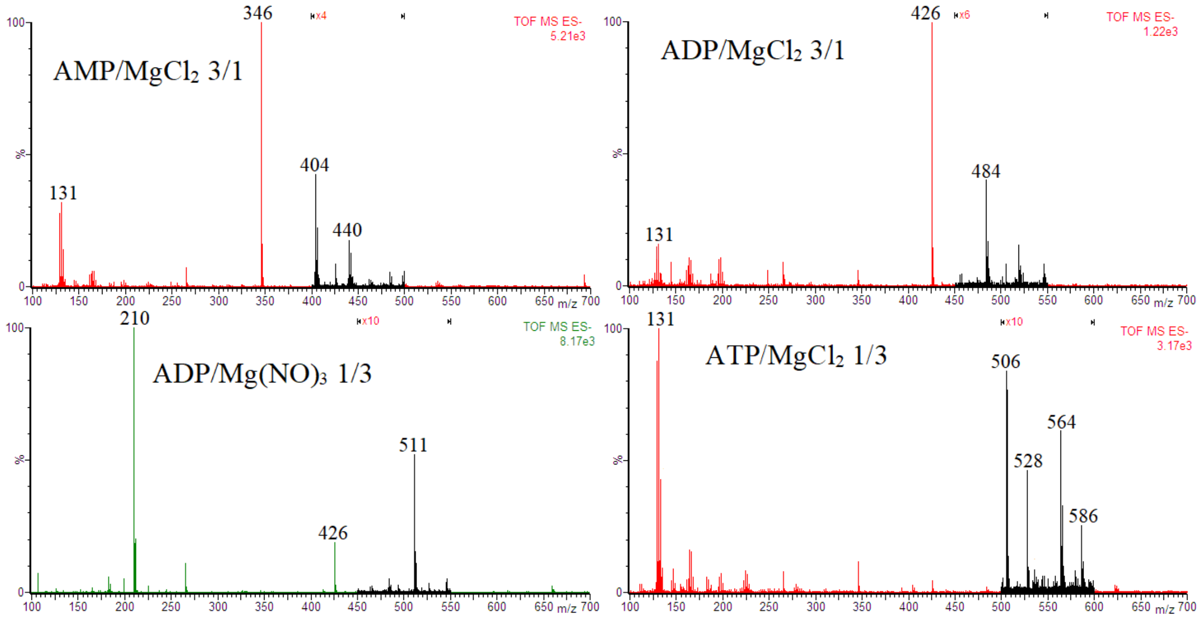

Figure S1. Exemplary full scan ESI mass spectra obtained for methanol/water solutions containing nucleotide and $MgCl_2$ or $Mg(NO_3)_2$. $[MgCl_3]^-$ – *m/z* 131; $[Mg(NO_3)_3]^-$ – *m/z* 210; $[AMP\text{-}H]^-$ – *m/z* 346; $[AMP\text{-}2H\text{+}MgCl]^-$ – *m/z* 404; $[AMP\text{-}H\text{+}MgCl_2]^-$ – *m/z* 440; $[ADP\text{-}H]^-$ – *m/z* 426; $[ADP\text{-}2H\text{+}MgCl]^-$ – *m/z* 484; ; $[ADP\text{-}2H\text{+}MgNO_3]^-$ – *m/z* 511; $[ATP\text{-}H]^-$ – *m/z* 506; $[ATP\text{-}3H\text{+}Mg]^-$ – *m/z* 528; $[ATP\text{-}2H\text{+}MgCl]^-$ – *m/z* 564; $[ATP\text{-}4H\text{+}Mg_2Cl]^-$ – *m/z* 586.

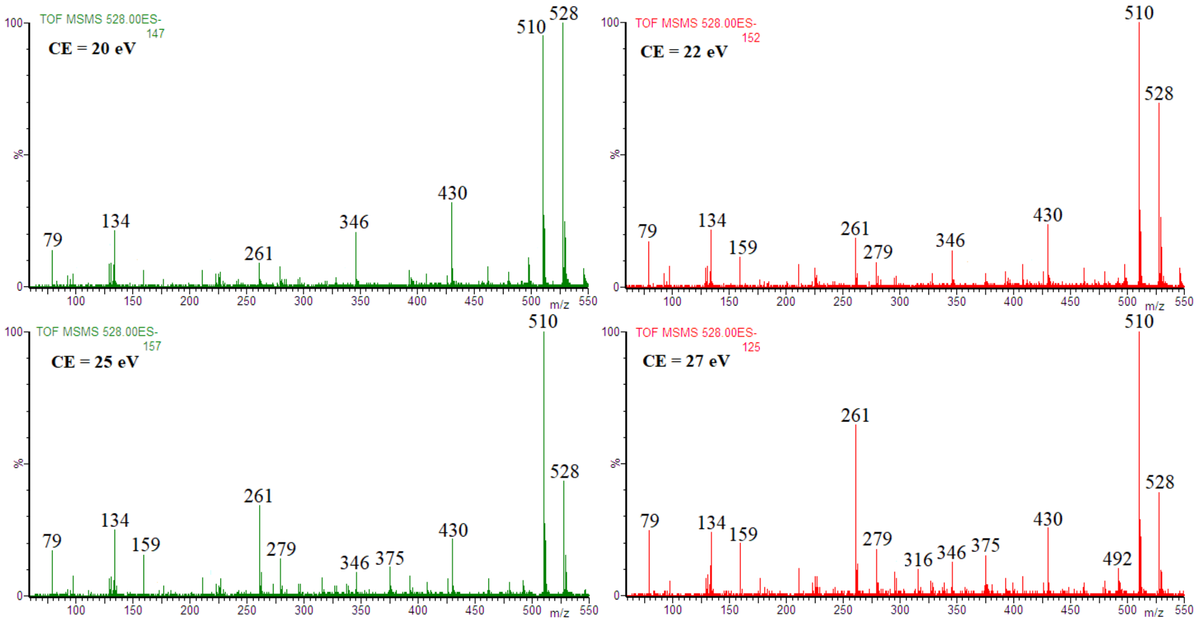

Figure S2. Product ion spectra of [ATP-3H+Mg]$^-$ ion – *m/z* 528. H_2O loss – *m/z* 510; H_3PO_4 loss – *m/z* 430; H_2O and adenine loss – *m/z* 375; MgP_2O_6 loss – m/z 346; **HPO_3 and ribose residue loss – *m/z* 316;** $[MgH_2P_3O_{10}]^-$ – *m/z* 279; $[MgP_3O_9]^-$ – *m/z* 261; $[HP_2O_6]^-$ – *m/z* 159; [adenine-H]$^-$ – *m/z* 134; PO_3^- – *m/z* 79.

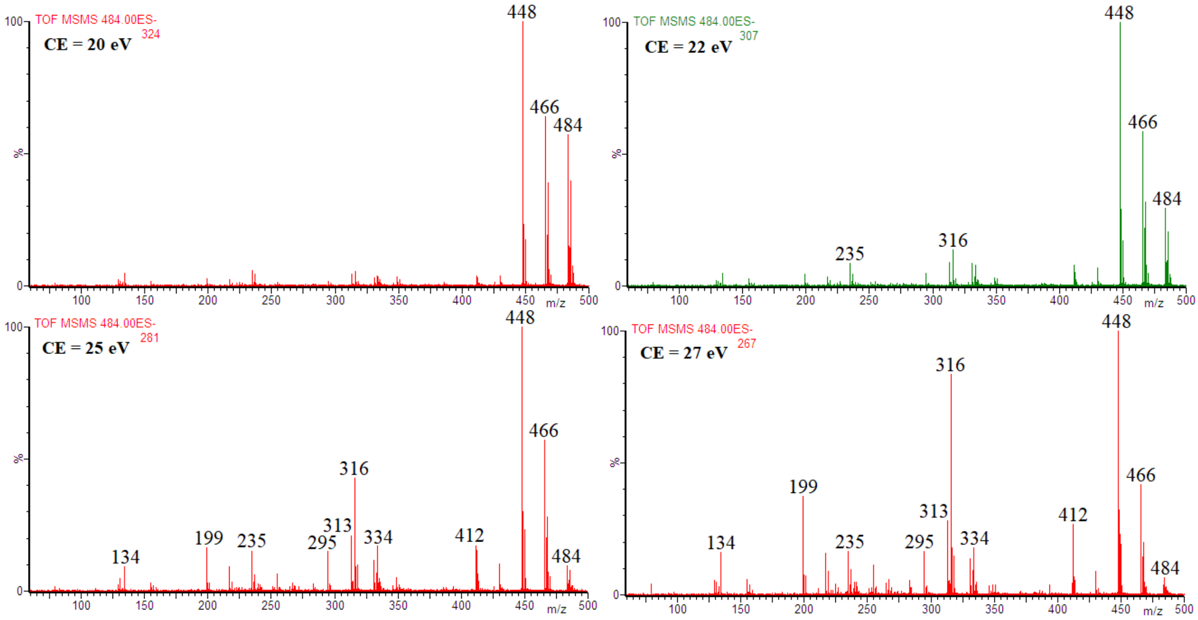

Figure S3. Product ion spectra of $[ADP\text{-}2H\text{+}MgCl]^-$ ion – *m/z* 484. H_2O loss – *m/z* 466; HCl loss – *m/z* 448; $2H_2O$ and HCl loss – *m/z* 412; H_2O and ribose residue loss – *m/z* 334; **HCl and ribose residue loss – *m/z* 316;** HCl and adenine loss – *m/z* 313; H_2O, HCl and adenine loss – *m/z* 295; $[MgClH_2P_2O_7]^-$ – *m/z* 235; $[MgHP_2O_7]^-$ – *m/z* 199; $[adenine\text{-}H]^-$ – *m/z* 134.

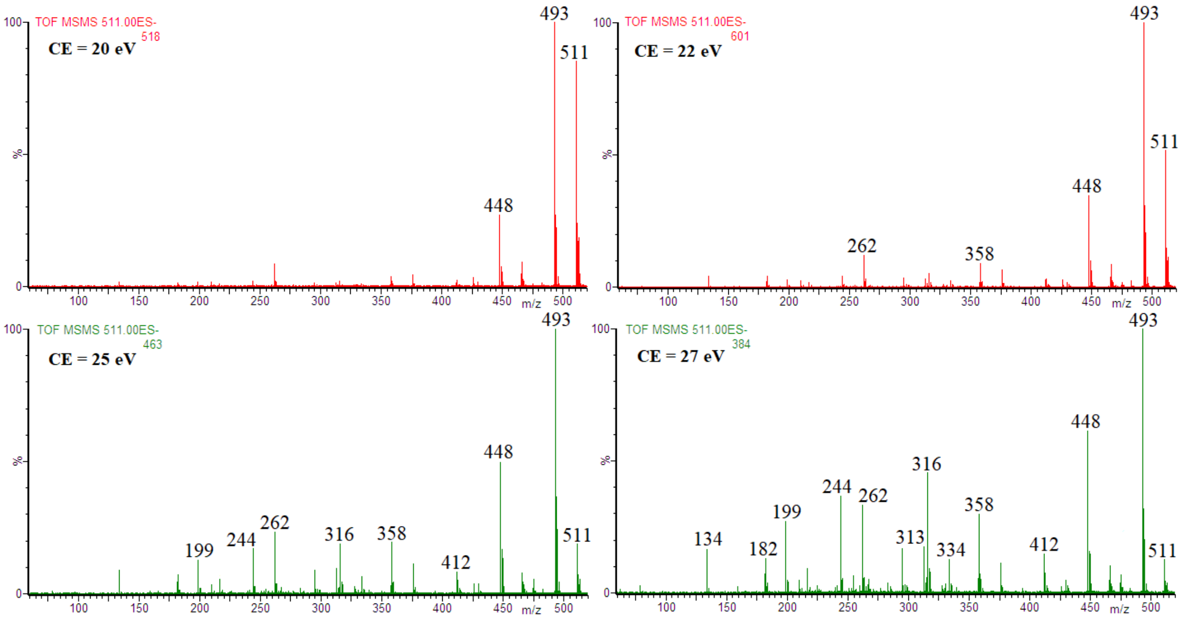

Figure S4. Product ion spectra of $[ADP\text{-}2H\text{+}MgNO_3]^-$ ion – *m/z* 511. H_2O loss – *m/z* 493; HNO_3 loss – *m/z* 448; $2H_2O$ and HNO_3 loss – *m/z* 412; H_2O and adenine loss – *m/z* 358; **HNO_3 and ribose residue loss – *m/z* 316;** HNO_3 and adenine loss – *m/z* 313; H_2O, HNO_3 and adenine loss – *m/z* 295; $[MgNO_3H_2P_2O_7]^-$ – *m/z* 262; $[MgNO_3P_2O_6]^-$ – *m/z* 244; $[MgHP_2O_7]^-$ – *m/z* 199; $[MgNO_3HPO_4]^-$ – *m/z* 182; $[adenine\text{-}H]^-$ – *m/z* 134.

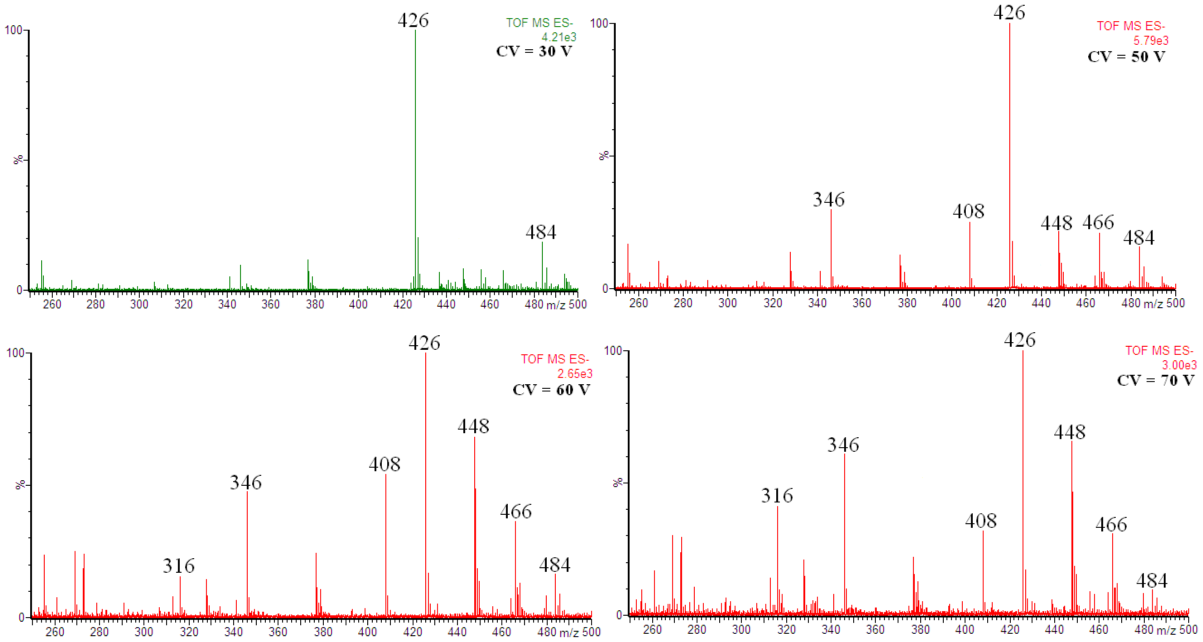

Figure S5. Full scan ESI mass spectra obtained for solution containing ADP and $MgCl_2$ at different cone voltages (CV). At higher cone voltages the abundant product ions of interest, namely at *m/z* 448 and 316 were generated, as a result of in-source fragmentation. $[ADP\text{-}2H\text{+}MgCl]^-$ ion – *m/z* 484; H_2O loss – *m/z* 466; **$[ADP\text{-}3H\text{+}Mg]^-$ ion (HCl loss) – *m/z* 448**; $[ADP\text{-}H]^-$ ion – *m/z* 426; $[ADP\text{-}H\text{-}H_2O]^-$ ion – *m/z* 408; $[ADP\text{-}H\text{-}HPO_3]^-$ ion ($[AMP\text{-}H]^-$ – *m/z* 346; **HCl and ribose residue loss from $[ADP\text{-}2H\text{+}MgCl]^-$ – *m/z* 316.**

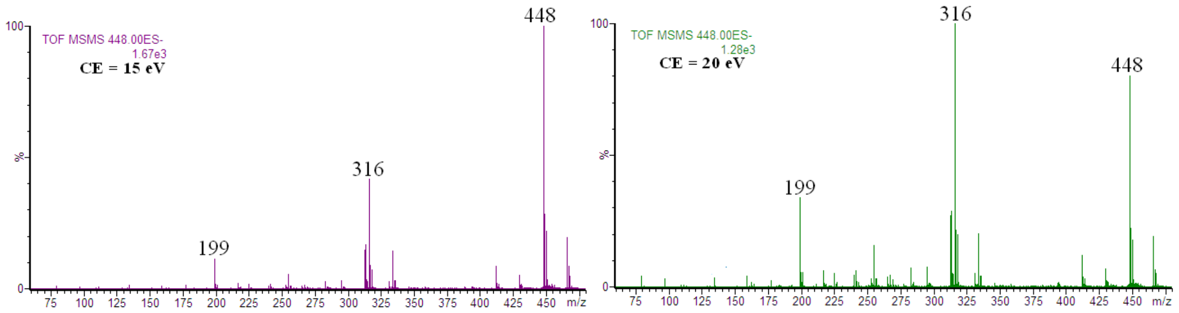

Figure S6. Product ion spectra of $[ADP\text{-}3H\text{+}Mg]^-$ ion – *m/z* 448 (ion generated at high cone voltage). Abundant product ion at *m/z* 316 was formed.

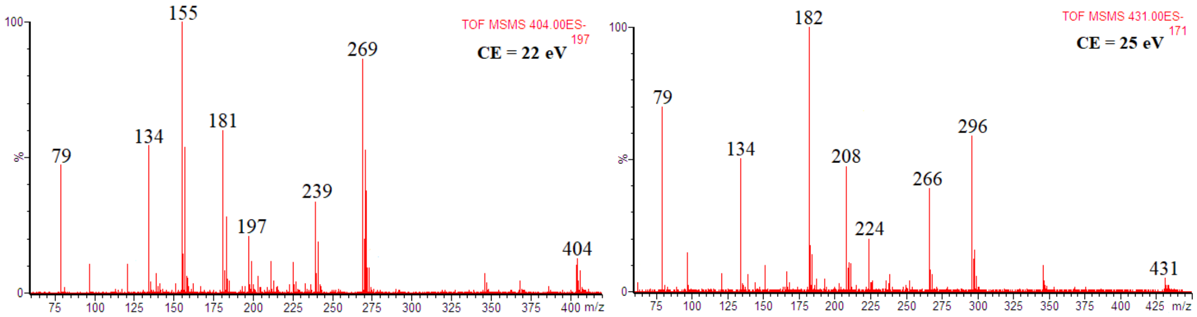

Figure S7. Product ion spectra of $[AMP\text{-}2H+MgCl]^-$ ion – *m/z* 404 and $[AMP\text{-}2H+MgNO_3]^-$ ion – *m/z* 431. Adenine loss – *m/z* 269 and 296, respectively; adenine and H_2CO loss – *m/z* 239 and 266, respectively; $[MgNO_3PO_4CH_2CHO_2]^-$ – *m/z* 224; $[MgNO_3PO_4CH_2CHO]^-$ – *m/z* 208; $[MgClPO_4CH_2CHO_2]^-$ – *m/z* 197; $[MgClPO_4CH_2CHO]^-$ – *m/z* 181; $[MgHPO_4+NO_3]^-$ – *m/z* 182; $[MgHPO_4+Cl]^-$ – *m/z* 155; [adenine-H]$^-$ – *m/z* 134; PO_3^- – *m/z* 79; (formation of the product ions at *m/z* 224, 182, 197, 181 occurs as a result of the breaking of ribose moiety).

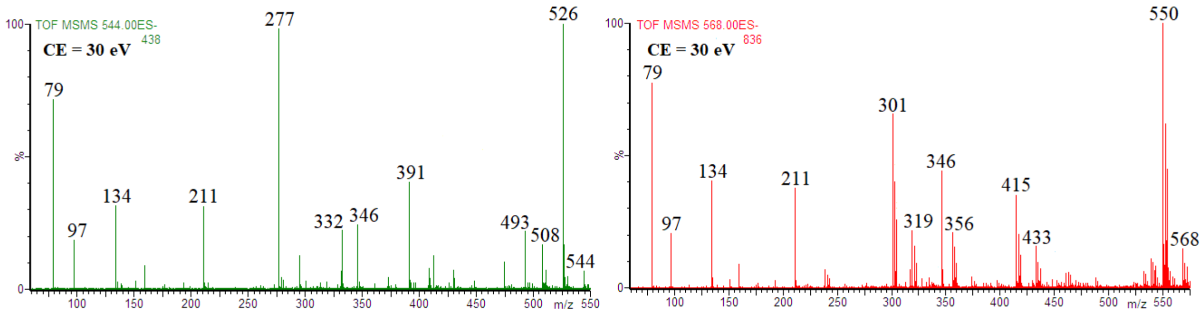

Figure S8. Product ion spectra of $[ATP\text{-}3H+Ca]^-$ ion – *m/z* 544 and $[ATP\text{-}3H+Zn]^-$ ion – *m/z* 568. H_2O loss – *m/z* 526 and 550, respectively; H_2O and adenine loss – *m/z* 391 and 415, respectively; **HPO_3 and ribose residue loss – *m/z* 332 and 356 respectively;** $[AMP\text{-}H]^-$ – *m/z* 346; $[CaP_3O_9]^-$ – *m/z* 277; CaP_2O_6 or ZnP_2O_6 and adenine loss – *m/z* 211; $[adenine\text{-}H]^-$ – *m/z* 134; $H_2PO_4^-$ – *m/z* 97; PO_3^- – *m/z* 79.

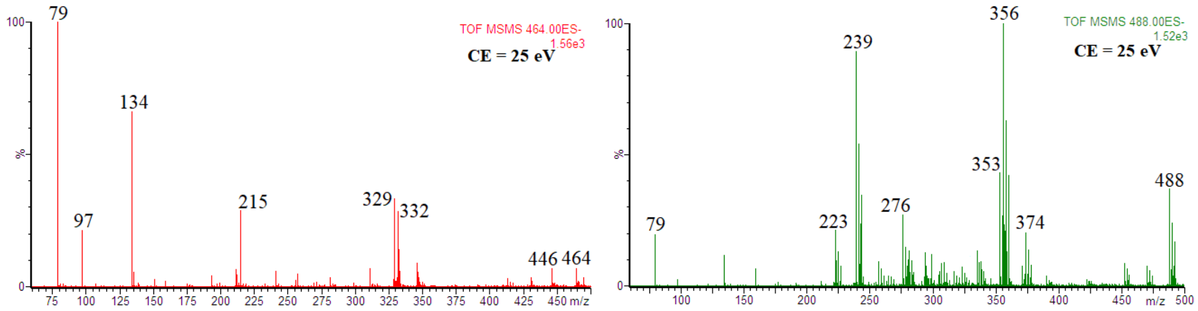

Figure S9. Product ion spectra of $[ADP\text{-}3H+Ca]^-$ ion – *m/z* 464 and $[ADP\text{-}3H+Zn]^-$ ion – *m/z* 488. **Ribose residue loss – *m/z* 332 and 356 respectively;** adenine loss – *m/z* 329 and 353, respectively; $[CaHP_2O_7]^-$ – *m/z* 215; $[ZnHP_2O_7]^-$ – *m/z* 239; HPO_3 and ribose residue loss – *m/z* 276; $[adenine\text{-}H]^-$ – *m/z* 134; $H_2PO_4^-$ – *m/z* 97; PO_3^- – *m/z* 79.

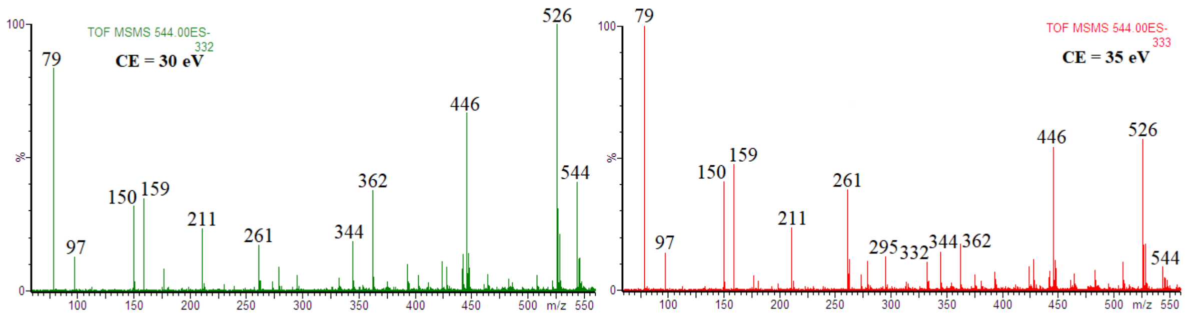

Figure S10. Product ion spectra of [GTP-3H+Mg]$^-$ ion – *m/z* 544. H_2O loss – *m/z* 526; H_3PO_4 loss – *m/z* 446; [GMP-H]$^-$ – *m/z* 362; [GMP-H-H_2O]$^-$ – *m/z* 344; **HPO_3 and ribose residue loss – *m/z* 332;** [MgP_3O_9]$^-$ – *m/z* 261; MgP_2O_6 and guanine loss – *m/z* 211; [HP_2O_6]$^-$ – *m/z* 159; [guanine-H]$^-$ – *m/z* 150; PO_3^- – *m/z* 79.

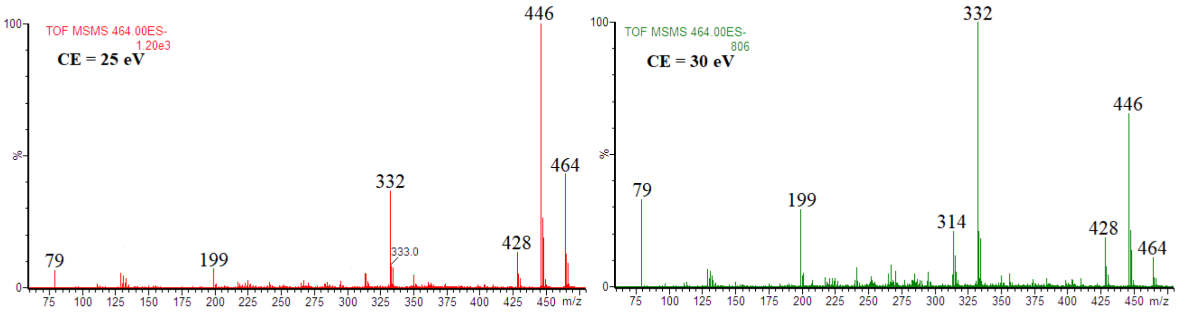

Figure S11. Product ion spectra of [GDP-3H+Mg]$^-$ ion – *m/z* 464. H_2O loss – *m/z* 446; $2H_2O$ loss – *m/z* 428; **ribose residue loss – *m/z* 332; H_2O and ribose residue loss – *m/z* 314;** [$MgHP_2O_7$]$^-$ – *m/z* 199; PO_3^- – *m/z* 79.

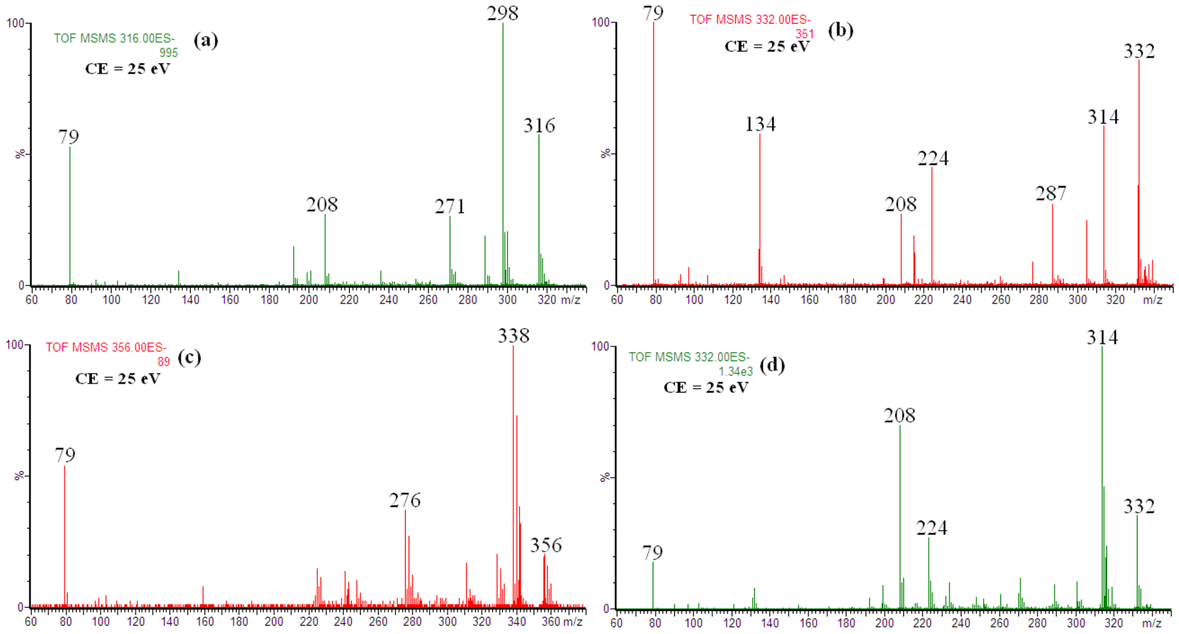

Figure S12. Product ion spectra of product ions formed as a results of internal ribose residue loss from studied complexes. **(a):** [(O_2POPO_3)Mg(adenine-H)]$^-$ – *m/z* 316, H_2O loss – *m/z* 298, H_2O and HCN loss – *m/z* 271, [(O_2POPO_3)MgCN]$^-$ – *m/z* 208, PO_3^- – *m/z* 79; **(b)**: [(O_2POPO_3)Ca(adenine-H)]$^-$ – *m/z* 332, H_2O loss – *m/z* 314, H_2O and HCN loss – *m/z* 287, [(O_2POPO_3)CaCN]$^-$ – *m/z* 224, [adenine-H]$^-$ – *m/z* 134, PO_3^- – *m/z* 79; **(c):** [(O_2POPO_3)Zn(adenine-H)]$^-$ – *m/z* 356, H_2O loss – *m/z* 338, HPO_3 loss – *m/z* 276, PO_3^- – *m/z* 79; **(d)**: [(O_2POPO_3)Mg(guanine-H)]$^-$ – *m/z* 332, H_2O loss – *m/z* 314, [(O_2POPO_3)MgCN]$^-$ – *m/z* 208, PO_3^- – *m/z* 79.

The following features of the above product ion spectra can be mentioned.

1. The signals corresponding to the loss of water molecule are features of all product ion spectra. It is reasonable to conclude that upon the loss of water molecule the P=N bond is formed.
2. Only for ion [(O_2POPO_3)Ca(adenine-H)]$^-$ the abundant product ion [adenine-H]$^-$ at *m/z* 134 was formed.
3. Only for ion [(O_2POPO_3)Zn(adenine-H)]$^-$ the loss of HPO_3 molecule was observed.
4. Formation of product ions [(O_2POPO_3)MgCN]$^-$, [(O_2POPO_3)CaCN]$^-$ (*m/z* 208 and 224 respectively) requires breaking of adenine skeleton, thus, it indicates the strong interaction between the metal cation and adenine (guanine) moiety.

Table S1. Atomic coordinates of structures A1 and A2 of [ADP-3H+Mg]$^{-}$ ion.

	Structure A1			Structure A2		
Atom	x	y	z	x	y	z
N	-2.48750	-4.24531	0.60299	-2.03712	2.57471	-0.73082
C	-3.04315	-3.04343	0.32246	-0.86965	2.41639	-0.10677
C	-2.30654	-1.86478	0.07702	0.37018	2.48818	-0.66892
N	-0.94803	-1.51310	0.05571	0.76220	2.76725	-1.88998
C	-0.94393	-0.22734	-0.27452	1.90926	2.03325	-1.95032
N	-2.16704	0.28986	-0.42841	2.24628	1.51727	-0.71684
C	-2.30626	1.70511	-0.81348	3.33613	0.55407	-0.46510
O	-1.12491	2.03985	-1.51493	3.12855	-0.59972	-1.40287
C	-0.27761	2.90701	-0.68027	2.56042	-1.69883	-0.54263
C	1.24391	2.79472	-0.86204	1.47512	-2.66014	-1.18412
O	2.01193	1.57626	-0.98188	0.18365	-2.16857	-1.62201
P	2.62562	0.95556	0.41331	-0.86718	-1.91385	-0.47658
O	1.48337	0.08312	1.01757	0.04823	-1.14676	0.71269
O	3.26838	2.00341	1.25998	-1.44043	-3.19581	0.06483
O	3.81075	-0.12124	-0.13072	-2.12624	-1.00766	-0.98564
P	3.41125	-1.67698	-0.38603	-3.43759	-0.25040	-0.00997
O	2.80244	-2.47633	0.85700	-3.08046	-0.35725	1.66205
O	4.71204	-2.57371	-0.38433	-3.57748	1.29504	-0.43103
O	2.27795	-1.77954	-1.43202	-4.74722	-0.94275	-0.26618
C	-0.85380	2.68139	0.72638	2.01640	-0.96594	0.67501
O	-0.52038	3.65102	1.68300	1.67077	-1.82987	1.75978
C	-2.32840	2.67599	0.37873	3.20719	-0.07915	0.92355
O	-2.65982	3.96686	-0.09422	4.35980	-0.87022	1.23900
C	-3.07858	-0.72257	-0.20383	1.42432	1.99840	0.13340
N	-4.39979	-0.64321	-0.24805	1.37019	2.07425	1.63863
C	-4.97308	-1.81074	-0.00073	0.23217	2.61498	1.73927
N	-4.38414	-2.97592	0.26695	-0.57848	1.98752	0.99288
H	-3.09483	-5.02813	0.75669	-2.13916	3.30999	-1.39931
H	-1.49762	-4.37490	0.66002	-2.82932	1.91965	-0.57265
H	-3.20753	1.77676	-1.42377	2.47218	1.87791	-2.84588
H	-0.52000	3.93206	-0.97639	4.26387	1.07382	-0.57932
H	1.79903	3.67668	-1.18497	3.38873	-2.30944	-0.24632
H	1.44853	3.04542	0.20722	1.59757	-3.72241	-1.10301
H	-0.57053	1.69389	1.09957	1.07950	-2.77541	-0.19625
H	0.42568	3.61857	1.86582	1.14878	-0.38841	0.43985
H	-3.00177	2.39462	1.19423	0.93946	-2.39352	1.49170
H	-3.56840	3.97768	-0.40321	3.07597	0.63521	1.70996
H	-6.05310	-1.82243	-0.03571	5.13385	-0.30159	1.29853
H	-0.07871	0.32089	-0.40048	0.01729	3.50146	2.29842
Mg	1.06212	-1.69365	0.18882	-1.27009	0.16423	1.23168

Table S2. Atomic coordinates of structures A3 and A4 of [ADP-3H+Mg]$^{-}$ ion.

	Structure A3			Structure A4		
Atom	x	y	z	x	y	z
N	3.88710	-0.13427	0.06361	-1.08122	2.95812	-0.11730
C	3.20385	-1.18425	0.42881	-0.04333	2.29419	0.27611
C	2.35622	-1.67336	-0.51619	1.14962	2.40989	-0.30153
N	2.43642	-2.05275	-1.79513	1.58485	3.13102	-1.36428
C	1.14288	-2.34326	-2.09332	2.68866	2.42569	-1.70960
N	0.36226	-2.06654	-1.00706	2.75395	1.44270	-0.82043
C	-1.11090	-2.23862	-0.97508	3.45971	0.17386	-0.80566
O	-1.77896	-1.05202	-1.55931	2.56474	-0.75776	-1.56982
C	-2.84894	-0.73774	-0.62172	2.25592	-1.84776	-0.64868
C	-3.50871	0.64401	-0.87308	0.98796	-2.72972	-1.06373
O	-2.71587	1.82431	-1.03607	-0.34920	-2.22448	-1.45780
P	-1.51374	1.96465	-0.10457	-1.51392	-1.80264	-0.53765
O	-1.70443	0.70607	1.06729	-0.94630	-1.74238	1.04173
O	-1.52547	3.29411	0.60580	-2.65709	-2.77343	-0.65630
O	-0.12680	1.88848	-0.98997	-2.14014	-0.22322	-0.95568
P	1.52504	2.20757	-0.38079	-3.62574	0.40535	-0.16311
O	1.34207	2.13047	1.31676	-3.51698	0.31794	1.56430
O	2.70118	1.19869	-0.99464	-3.76188	2.04593	-0.62540
O	1.94718	3.57585	-0.78458	-4.84515	-0.35338	-0.61235
C	-2.10921	-0.89362	0.70442	2.13361	-1.07656	0.67617
O	-2.93676	-0.68720	1.87064	1.94335	-1.90208	1.82870
C	-1.70819	-2.33137	0.43853	3.49506	-0.41942	0.61175
O	-2.92183	-3.07599	0.35942	4.47802	-1.45533	0.68895
C	1.19823	-1.67293	-0.10004	1.97678	1.72243	0.10912
N	1.23458	-1.12569	1.21824	2.02501	1.26124	1.49710
C	2.03367	-1.55423	2.12634	0.88152	1.14983	2.02563
N	3.22811	-1.71502	1.63291	-0.08483	1.38843	1.22914
H	4.85000	0.02056	0.28118	-1.96489	2.81584	0.32950
H	3.23605	0.59827	-0.51846	-2.98607	2.52774	-0.33038
H	0.79843	-2.72584	-3.04100	3.34830	2.60752	-2.53335
H	-1.32211	-3.14891	-1.49634	4.45337	0.27669	-1.18791
H	-3.63831	-1.46470	-0.66591	3.08312	-2.52338	-0.58044
H	-4.56342	0.75062	-0.74575	1.05717	-3.78481	-0.90121
H	-3.49639	0.76284	0.19606	0.70479	-2.73137	-0.03117
H	-1.27217	-0.23443	0.79201	1.33197	-0.36825	0.65559
H	-3.24343	0.22937	1.88510	1.09646	-2.35015	1.76404
H	-1.05584	-2.80371	1.15188	3.71022	0.30049	1.37384
H	-2.72199	-3.94949	0.01920	5.35136	-1.07831	0.56033
H	1.78904	-1.69394	3.15494	0.73828	0.82198	3.03472
Mg	0.19130	0.51883	1.29372	-1.60680	0.09213	1.36667

Table S3. Atomic coordinates of structures B1, B2 and B3 of $[(O_2POPO_3)Mg(adenine\text{-}H)]^-$ ion.

Atom	B1			B2			B3		
	x	y	z	x	y	z	x	y	z
P	2.56676	1.67658	-0.31043	2.78832	1.51222	-0.60241	3.18101	1.37429	-0.55016
P	2.96106	-1.25694	0.26575	2.73027	-1.22616	0.62833	2.54892	-1.44755	0.45139
O	3.56426	0.52903	-0.06725	3.59323	0.38421	0.07588	3.72525	0.00028	-0.15099
O	3.06153	3.05390	-0.53623	3.51085	2.71209	-1.09183	4.05930	2.35051	-1.23271
O	1.08844	1.32226	-0.31100	1.29001	1.33750	-0.72324	1.73025	1.68165	-0.21045
O	4.25369	-1.96748	0.46171	3.89240	-2.01603	1.12900	3.59702	-2.46123	0.77366
O	1.95595	-0.93238	1.41786	1.64633	-0.60129	1.55983	1.78652	-0.72432	1.61912
O	2.05312	-1.41986	-0.99587	1.97357	-1.53289	-0.69824	1.60083	-1.39954	-0.76730
Mg	0.52431	-0.61353	0.03857	0.40421	-0.38850	-0.03748	0.53225	0.15484	0.39876
N	-1.45744	-0.91094	-0.15851	-3.61799	-1.10078	-1.53832	-4.52102	0.62757	1.22985
C	-1.97707	-2.13604	-0.45351	-2.37014	-1.45222	-1.83865	-3.60514	1.01725	2.12518
N	-3.29565	-2.23147	-0.49980	-1.38463	-0.94573	-1.04218	-2.28955	0.87766	1.78875
C	-2.59185	-0.13584	-0.00173	-3.44775	-0.28556	-0.44111	-3.73786	0.17141	0.20863
C	-3.71364	-0.95931	-0.20968	-2.08709	-0.20589	-0.15558	-2.38873	0.33089	0.57007
C	-2.82339	1.21436	0.30046	-4.27548	0.43427	0.43207	-4.00340	-0.37833	-1.05047
N	-4.08205	1.64745	0.39463	-3.72821	1.12268	1.44937	-2.99026	-0.77952	-1.84349
C	-5.07327	0.75906	0.18570	-2.39627	1.11079	1.60525	-1.74904	-0.60752	-1.40174
N	-4.98711	-0.52251	-0.11863	-1.50746	0.47738	0.85067	-1.37227	-0.05528	-0.24787
N	-1.81853	2.11751	0.55282	-5.62797	0.48521	0.27433	-5.27383	-0.51358	-1.49812
H	-2.13834	3.07235	0.57987	-6.16282	0.83639	1.04917	-5.44238	-0.90218	-2.40677
H	-0.92062	1.97589	0.11703	-6.04688	-0.18798	-0.34372	-6.02520	-0.20150	-0.91059
H	-1.32645	-2.98550	-0.64231	-2.13807	-2.10716	-2.67002	-3.88410	1.41945	3.09201
H	-6.07864	1.16770	0.27819	-2.00868	1.68141	2.44533	-0.94416	-0.95394	-2.04283

Table S4. Atomic coordinates of structure B4 and B5 of $[(O_2POPO_3)Mg(adenine\text{-}H)]^-$ ion.

Atom	B4			B5		
	x	y	z	x	y	z
P	3.33409	-1.35499	-0.40258	2.94831	1.55410	-0.45328
P	2.52433	1.45425	0.30440	2.82118	-1.33573	0.37923
O	3.74625	0.12676	-0.26724	3.72965	0.28482	-0.05512
O	4.35530	-2.36433	-0.75874	3.69655	2.77101	-0.85023
O	1.87210	-1.69108	-0.16798	1.43453	1.49026	-0.40067
O	3.39120	2.65940	0.37667	3.96812	-2.25923	0.60751
O	1.42792	1.23820	-0.79713	1.90804	-0.78847	1.52353
O	1.95476	0.70284	1.55173	1.88211	-1.39594	-0.86566
Mg	0.61400	-0.19738	0.36263	0.51742	-0.27143	0.14259
N	-4.57895	1.20090	-0.62528	-4.29741	0.98587	1.24897
C	-5.63390	0.41118	-0.39516	-5.46404	0.70222	0.65483
N	-5.40665	-0.82780	0.11243	-5.45829	-0.07806	-0.45381
C	-3.55189	0.39488	-0.22342	-3.42285	0.31934	0.44279
C	-4.06676	-0.84828	0.22624	-4.14876	-0.34014	-0.61360
C	-2.17598	0.59567	-0.20529	-2.05872	0.15655	0.43310
N	-1.39432	-0.41784	0.25061	-1.46510	-0.58022	-0.52132
C	-2.00075	-1.58143	0.65443	-2.23796	-1.15908	-1.46789
N	-3.27109	-1.86309	0.67503	-3.54956	-1.08078	-1.56830
N	-1.60961	1.76783	-0.59438	-1.10567	0.70242	1.36541
H	-0.62594	1.83101	-0.82303	-1.30613	0.38332	2.30820
H	-2.24203	2.43100	-1.01042	-1.16915	1.71586	1.38441
H	-6.64435	0.74749	-0.60767	-6.39806	1.08921	1.05191
H	-1.31217	-2.35615	0.99424	-1.70383	-1.74442	-2.21181

Table S5. Atomic coordinates of 1,5-anhydro-*β*-D-ribofuranose.

Atom	x	y	z
O	-1.74428	-0.84476	-0.32291
C	-1.86272	0.58203	-0.47522
C	-0.61317	1.08245	0.25760
O	-0.48702	0.08710	1.29086
C	-0.54037	-1.02687	0.41141
C	0.63115	-0.78470	-0.54972
O	1.81641	-1.31510	-0.00498
C	0.63324	0.78678	-0.58320
O	1.79892	1.31427	0.02105
H	-1.87062	0.84845	-1.53723
H	-2.78407	0.91807	0.00785
H	-0.66113	2.08428	0.68274
H	-0.54562	-1.96999	0.95349
H	0.41798	-1.22054	-1.53144
H	2.52590	-0.68453	-0.17032
H	0.58636	1.19711	-1.59368
H	1.77018	1.05693	0.95111
